# Akzeptanz psychosozialer Überbrückungsmaßnahmen im Rahmen demenzieller Erkrankungen: Behandlungsbeobachtung während der COVID-19-Pandemie

**DOI:** 10.1007/s00391-022-02115-6

**Published:** 2022-10-07

**Authors:** V. Buschert, B. Leicher, C. Rogl, J. Hoffmann, A.-L. Blum, N. Scherbaum, J. Benninghoff

**Affiliations:** 1 Zentrum für Altersmedizin und Entwicklungsstörungen, kbo-Isar-Amper-Klinikum München-Ost, Ringstr. 52, 85540 Haar, Deutschland; 2 Klinik für Psychiatrie und Psychotherapie, LVR-Klinikum Essen, Kliniken und Institut der Universität Duisburg-Essen, Wickenburgstraße 21, 45147 Essen, Deutschland

**Keywords:** Belastung, Kognitives Training, Unterstützungsmaßnahmen, Behandlungsausfall, Angehörigenarbeit, Burden, Cognitive training, Support, Temporary cessation of treatment, Caregiver support

## Abstract

**Hintergrund:**

Bei COVID-19 bedingtem Ausfall eines ambulanten Behandlungsprogramms im Rahmen demenzieller Erkrankungen wurden Patient:innen und deren Angehörigen Überbrückungsmaßnahmen auf Distanz angeboten. Ziel war es zu untersuchen, in welchem Maß die Teilnehmer:innen (TN) belastet waren, und wie die Angebote angenommen und bewertet wurden.

**Material und Methoden:**

Allen TN (*n* = 63) wurden über den Zeitraum von 8 Wochen regelmäßige Telefonkontakte in unterschiedlicher Frequenz (wöchentlich, 14-tägig) sowie die Zusendung kognitiver und körperlicher Trainingsaufgaben im Abstand von 2 Wochen angeboten. Um die Akzeptanz der Maßnahmen sowie das Belastungserleben der TN zu erfassen, wurden aus der klinischen Routine erhobene Daten sowie retrospektiv eine schriftliche Befragung der TN in eine Behandlungsbeobachtung einbezogen.

**Ergebnisse:**

Von 63 kontaktierten TN wurden die Daten von 45 TN eingeschlossen. Die Überbrückungsmaßnahmen wurden durchwegs positiv bewertet, wobei eine tendenziell höhere Zustimmung vonseiten der Angehörigen zu ermitteln war. Bei allen TN blieb das Belastungserleben stabil auf geringem Niveau; Angehörige waren signifikant stärker belastet.

**Diskussion:**

Die Ergebnisse der vorliegenden Behandlungsbeobachtung sprechen für eine gut durchführbare und ortsunabhängige psychosoziale Behandlungsform im Rahmen demenzieller Erkrankungen. Diese kann sowohl als Überbrückungsmaßnahme für zukünftige pandemiebedingte Behandlungsausfälle als auch für die Routineversorgung (z. B. für mobilitätseingeschränkte oder im ländlichen Raum ohne direkte Klinikanbindung lebende Betroffene) geeignet sein. Daneben zeigt die Studie, wie notwendig es ist, für Angehörige ebenfalls Maßnahmen zu prüfen, um die Belastung zu reduzieren.

**Zusatzmaterial online:**

Zusätzliche Informationen sind in der Online-Version dieses Artikels (10.1007/s00391-022-02115-6) enthalten.

## Abstract (en)

**Background:**

In the event of a COVID-19-related absence from an outpatient treatment program, patients suffering from dementia and their caregivers were offered support from a distance. The aim was to examine the extent of the participants’ burden as well as how the offer was accepted and evaluated.

**Material and methods:**

All participants (*n* = 63) were offered supportive telephone contact over a period of 8 weeks at varying frequencies (weekly, fortnightly). In addition patients received cognitive and physical tasks by mail every 2 weeks. In order to examine the acceptance of the support, data collected from clinical routine were included in a treatment observation. Additionally, all participants were asked to evaluate the support retrospectively.

**Results:**

Out of 63 contacted participants, 45 were included in the treatment observation. The telephone support was very well accepted and a tendency towards higher agreement could be ascertained by caregivers. The experience of burden remained stable at a moderate level for all participants; however, caregivers were significantly more burdened.

**Conclusion:**

The present treatment observation shows the acceptance of a location-independent psychosocial treatment program for dementia for future pandemics or treatment failure as well as for the routine care, e.g. for patients with restricted mobility or those living in rural areas without direct hospital connection. In particular, our data strengthen the importance of programs for caregivers in order to alleviate their burden.

Im Zuge der weltweiten Ausbereitung von COVID-19 kam es zu umfangreichen Beschränkungen für die Allgemeinbevölkerung, die einen massiven Einschnitt in das alltägliche Leben darstellten und als außergewöhnliche und belastende Lebensereignisse verstanden werden können [13]. Aus zahlreichen Studien ergeben sich Hinweise auf negative psychische Folgen im Zuge von Quarantänemaßnahmen und Ausgangs- und Kontaktbeschränkungen bei schwerwiegenden COVID-19-Ausbrüchen [10].

Ältere Menschen mit demenziellen Erkrankungen gelten als Risikogruppe, schwer an COVID-19 zu erkranken oder daran zu versterben [2]. Darüber hinaus bergen restriktive Maßnahmen der sozialen Kontakt- und Ausgangsbeschränkungen gerade für die ältere Bevölkerung die Gefahr erheblicher Folgen auf körperlicher, sozialer, kognitiver, emotionaler und versorgungsbezogener Ebene [1, 11]. Neben strukturellen Defiziten und Schwachstellen in der Gesundheitsversorgung und Pflege älterer Menschen [12] wird durch das Aufbrechen von gewohnten Tagesabläufen, fehlenden sozialen Kontakten, Wegfall von Unterstützungsangeboten, Abstandsregelungen usw. vor allem bei demenziell erkrankten Menschen auch die gesellschaftliche Teilhabe erschwert, was einen beschleunigten Progress der Symptomatik zur Folge haben kann.

Die Gruppe der pflegenden Angehörigen ist ebenfalls durch die COVID-19-bedingten Veränderungen besonders betroffen und belastet, wie u. a. eine bundesweite Befragung von 1000 Personen im Alter zwischen 40 und 85 Jahren vom Mai 2020 zeigt [8]. Deshalb forderten Fachgesellschaften und Experten aus der Alters- und Demenzforschung, demenziell Erkrankte nicht mehr in eine „krank machende Isolation“ zu schicken, sondern einen sicheren Normalbetrieb unter Beachtung der Hygieneregeln zu ermöglichen [7]. Psychologische Interventionen könnten hierbei einen Beitrag leisten, um negative Auswirkungen der COVID-19-Pandemie zu reduzieren und daraus für künftige derartige Ereignisse zu lernen [10].

Am Memory Zentrum (MZ) des Zentrums für Altersmedizin und Entwicklungsstörungen (ZfAE) des kbo-Isar-Amper Klinikums München-Ost wird seit Januar 2019 das „Aktiv+++-Programm“ für Menschen mit erworbenen kognitiven Einbußen mit beginnender oder bei manifester demenzieller Erkrankung unterschiedlicher Schweregrade angeboten [6]. Die Patient:innen (Pat.) nehmen wöchentlich an einem 50-minütigen (min) ambulanten kognitiven Gruppentraining/einer Stimulation sowie einer körperlichen Aktivierung von 50 min teil. Zeitgleich und in separaten Gruppen erhalten Angehörige (Ang.) wöchentlich für 50 min eine Schulung. Aufgrund der COVID-19-bedingten Infektionsgefahr musste das Behandlungsprogramm Ende Februar/Anfang März 2020 für den Zeitraum von 14 Wochen ausgesetzt werden. Zur Überbrückung wurden allen Teilnehmer:innen (TN) eine telefonische Begleitung sowie Übungsaufgaben per Post auf freiwilliger Basis angeboten. Um die Akzeptanz der Maßnahmen sowie das Belastungserleben der TN überprüfen zu können, wurden aus der klinischen Routine erhobene Daten in eine Behandlungsbeobachtung einbezogen. Anlass für diese „Fernbehandlung“ war die COVID-19-Pandemie; therapeutische Maßnahmen auf Distanz sind aber darüber hinaus sinnvoll und möglich, um ambulante Therapieangebote z. B. bei Mobilitätseinschränkungen oder auch im ländlichen Raum ohne direkte Klinikanbindung nutzen zu können.

## Methode

### Beobachtungsdesign

**Figure 1:**
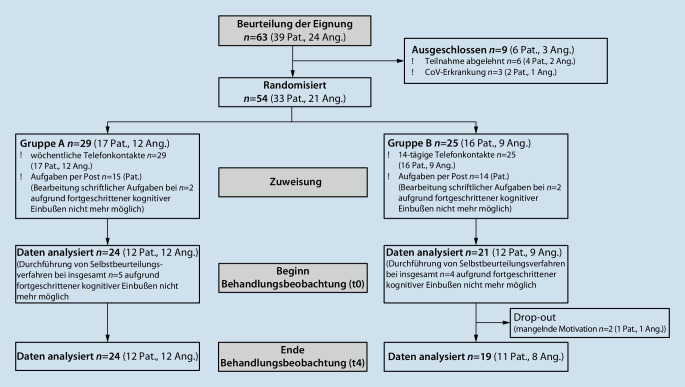

Bei der vorliegenden Untersuchung handelt es sich um eine Behandlungsbeobachtung mit 5 Beobachtungszeitpunkten (t0–t4) von ambulanten Patient:innen und – sofern vorhanden – deren Angehörigen (Abb. 1). Aufgrund staatlicher Maßnahmen musste das Aktiv+++-Programm ab dem 16.03.2020 ausgesetzt werden. Es wurde ein Überbrückungsprogramm konzipiert und mit einer jeweils 2‑wöchigen Vor- und Nachbereitungsphase vom 30.03.2020 bis zum Abklingen der ersten „Lockdown“-Phase allen TN der ambulanten Gruppen und zusätzlich Angehörigen, die bisher keine TN der Angehörigenschulung waren, bis 29.05.2020 angeboten und durchgeführt. Die Überbrückungsmaßnahmen bestanden für alle TN aus einer systematischen telefonischen Begleitung sowie für Patient:innen aus regelmäßig per Post zugesandten kognitiven und körperlichen Trainingsaufgaben. Aufgrund begrenzter personeller Kapazitäten und im Hinblick auf zukünftige Maßnahmen wurden die TN anhand randomisierter Zuteilung in unterschiedlicher Frequenz telefonisch kontaktiert: Gruppe A wöchentlich, Gruppe B 14-tägig.

Gegenstand der Behandlungsbeobachtung war die Überprüfung der Akzeptanz dieser Überbrückungsmaßnahmen sowie des Belastungserlebens der TN zu Beginn, während und kurz vor Wiederaufnahme des regulären Behandlungsprogramms. Dazu wurden im Rahmen der üblichen klinischen Routine eingesetzte Standardverfahren (Mini-Symptom-Checklist [Mini-SCL], Berliner Inventar zur Angehörigenbelastung-Demenz-Manual zur Praxisversion [BIZA-D-PV], subjektive Belastungserlebensskala [BS]) herangezogen und die Bewertung der Maßnahmen mit einem Abschlussfragebogen erhoben. Zugrunde lag die Genehmigung der zuständigen Ethikkommission der Medizinischen Fakultät der Universität Duisburg-Essen (AZ 20-9407-BO).

### Beobachtungsstichprobe

#### Einschlusskriterien und Datenerhebung

Von insgesamt 63 Aktiv+++-TN (39 Pat., 24 Ang.) wurden 54 TN (33 Pat., 21 Ang.–12 Dyaden und 12 Pat. ohne Ang.) in das Überbrückungsprogramm einbezogen (Abb. 1). Die Datenerhebung erfolgte telefonisch an 4 Beobachtungspunkten im Zeitraum vom 30.03.2020 bis 29.05.2020 (t0–t3) jeweils im Abstand von etwa 2 Wochen sowie schriftlich in Form einer Abschlussbefragung im Zeitraum vom 01.06.2020 bis 14.06.2020 (t4). Auch Patient:innen im mittel- bis schwergradigen Stadium einer Demenz (*n* = 9) wurden telefonisch kontaktiert. Eine Erhebung eigenanamnestischer Angaben oder die Durchführung von Selbstbeurteilungsverfahren konnte aufgrund ausgeprägter kognitiver Beeinträchtigungen jedoch nicht erfolgen.

#### Telefonische Begleitung

**Figure 2:**
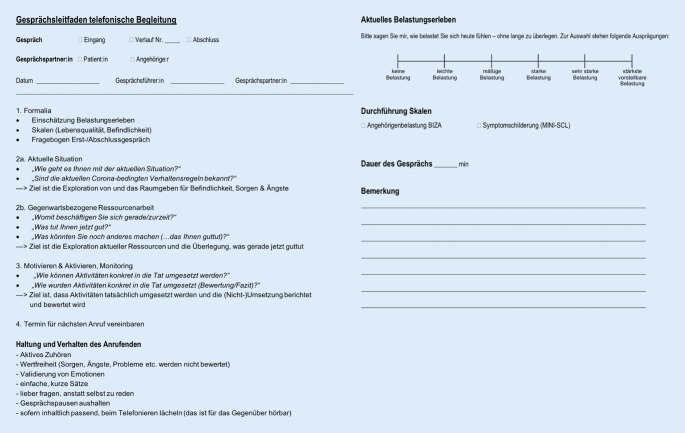

Bei der telefonischen Begleitung handelte es sich um supportive Maßnahmen mit stützenden Gesprächen zur emotionalen Stabilisierung, Aktivierung, Prävention sozialer Isolation sowie des Beziehungserhalts. Für die Telefonate wurden semistrukturierte Gesprächsleitfäden erstellt, die sich auf das subjektive Belastungserleben sowie Veränderungen hinsichtlich körperlicher, geistiger und sozialer Aktivitäten oder des Gesundheitszustands und auf die Bewertung der zugeschickten Aufgaben im Rückblick bezogen (Abb. 2). Wenn es die aktuelle Situation erforderte, wurden zudem psychoedukative und metakognitive Inhalte vermittelt.

#### Aufgaben per Post

Patient:innen mit Ausnahme derer, bei denen die Bearbeitung schriftlicher Aufgaben nicht mehr möglich war (*n* = 4), erhielten – unabhängig von der telefonischen Frequenz – 4‑mal im Abstand von 2 Wochen Aufgaben per Post. Die Aufgabenpakete beinhalteten 10 bis 12 Arbeitsblätter zur kognitiven Aktivierung z. B. aus den StaKogT/S-Programmen [4, 5] sowie Anleitungen zur körperlichen Aktivierung.

#### Zielparameter

Das Ziel der vorliegenden Behandlungsbeobachtung war, das subjektive Belastungserleben der TN während des Behandlungsausfalls zu erfassen sowie die Akzeptanz der Überbrückungsmaßnahmen zu überprüfen. Überdies war von Interesse, ob es Unterschiede hinsichtlich der Frequenz der telefonischen Kontakte gab.

#### Befragungsinstrumente

Zur Erfassung der Akzeptanz und Bewertung der Überbrückungsmaßnahmen wurde ein Abschlussfragebogen erstellt, in dem die Befragten Aussagen zu verschiedenen Themenbereichen (Zusatzmaterial online: Tab. 6) zustimmen („trifft eher zu“ und „trifft voll zu“) oder ablehnen („trifft eher nicht zu“ und „trifft gar nicht zu“) konnten. Der pseudonymisierte Fragebogen wurde den TN per Post zugesendet.

Für die Erhebung des subjektiven Belastungserlebens wurde eine 6‑stufige Rangskala (BS) erstellt (Abb. 2), die sich an herkömmlichen Skalen (z. B. zum Schmerzempfinden [16]) orientiert, sodass niedrige Werte ein geringes Belastungserleben darstellen (1: keine Belastung bis 6: stärkste vorstellbare Belastung). Aus der klinischen Routine wurde zudem die Mini-SCL [9] durchgeführt, die in einem Gesamtscore über 18 Items und 3 Subskalen (Depressivität, Ängstlichkeit, Somatisierung) das subjektive Beschwerdeerleben in abgestufter Ausprägung erfasst: Je höher der Wert, desto stärker das Beschwerdeerleben. Bei den Angehörigen wurden zusätzlich psychosoziale Belastungen und pflegebezogene Anforderungen in 5 Belastungsdimensionen (A: persönliche Einschränkungen; B: mangelnde soziale Unterstützung; C: Akzeptieren der Situation; D + E Verhaltensänderungen bei A (Kognition, Aggression und Verwirrtheit)) mit dem BIZA-D-PV [15] erhoben. Bei den Subskalen A bis C indizieren hohe Werte eine starke Belastung, bei den Subskalen D und E eine niedrige Belastung. Die Skalen wurden telefonisch abgefragt.

#### Datenerfassung

Die aus der klinischen Routine gesammelten Daten wurden nach Abschluss der ersten „COVID-19-Welle“ zum 01.08.2020 elektronisch, retrospektiv pseudonymisiert als Codes erfasst und ausgewertet. Damit können personenbezogene Daten ohne Hinzuziehung zusätzlicher Informationen nicht mehr einer spezifischen Person zugeordnet werden, wie es datenschutzrechtlich im Rahmen des Ethikvotums der medizinischen Fakultät der Universität Duisburg-Essen vorgegeben wurde.

#### Statistische Analysen

Alle Daten wurden mit dem Programm „Statistical Package for the Social Sciences (SPSS)“, Version 25.0 für Windows, bearbeitet. Soziodemografische Daten wurden mittels nichtparametrischer Verfahren (Chi-Quadrat und Likelihood-Ratio) verglichen. Für die Datenanalyse der Zielvariable „Akzeptanz der Überbrückungsmaßnahmen“ wurde vor dem Hintergrund der kleinen Gruppengrößen die Häufigkeiten des Abschlussfragebogens mittels Kreuztabellen berechnet.

Für die Zielvariablen „BS und Mini-SCL“ wurde eine multivariate Mixed-Model-Varianzanalyse mit BS- und Mini-SCL-Werten als abhängige Variablen zu je 5 Messzeitpunkten durchgeführt. Die unabhängigen Faktoren stellten dabei jeweils die Gruppe (wöchentlich vs. 14-tägig) und die Person (Pat. vs. Ang.) dar.

## Ergebnisse

### Soziodemografische und klinische Daten

Von 63 kontaktierten TN (24 Pat., 39 Ang.) wollten 6 TN (4 Pat., 2 Ang.) keine telefonische Begleitung in Anspruch nehmen, weitere 3 TN (2 Pat., 1 Ang.) entfielen aufgrund einer COVID-19-Erkrankung. Von 54 TN (33 Pat., 21 Ang.) wurden bei 9 Patient:innen im fortgeschrittenen Stadium einer Demenz keine eigenanamnestischen Angaben erhoben, sodass die Daten von 45 TN (24 Pat. und 21 Ang.) in die Behandlungsbeobachtung einflossen.

Die TN waren im Mittel 77,5 Jahre alt (SD = ± 8,5, min = 44,9, max = 92,3), zu 55,6 % weiblich und zu 44,4 % männlich. Angehörige waren bis auf eine Person ausnahmslos Ehepartner:innen. Einen Überblick über die demografischen und klinischen Daten bietet Tab. 1 (Zusatzmaterial online). Im Verlauf der telefonischen Begleitung schieden 2 TN (1 Pat., 1 Ang.) aus persönlichen Gründen aus (Drop-out: 4,7 %), sodass insgesamt 43 Personen die Behandlungsbeobachtung abschlossen.

### Umfang der Telefonkontakte

Die telefonische Begleitung umfasste insgesamt 176,3 h (10.577 min). Die durchschnittliche Gesprächsdauer pro TN über alle Telefonkontakte betrug 3,9 h (235,0 min, SD = ± 127,3 min), die durchschnittliche Gesprächsdauer pro Telefonkontakt 34 min (SD ± 12,9). Angehörige telefonierten im Durchschnitt signifikant länger als Patient:innen (28,7 min (SD = ± 12,3) vs. 40,1 min (SD = ± 10,9); *t* = −3,25*; p* *=* 0,002).

### Gruppen

Es gab keine signifikanten Unterschiede der Gruppen zu Beginn der Behandlungsbeobachtung hinsichtlich demografischer Daten (Alter, Geschlecht, Familien- oder Bildungsstand) sowie der Person (Patient:in vs. Angehörige/r); auch in klinischer Hinsicht (Schweregrad, Belastungserleben) gab es keine signifikanten Unterschiede (alle *p* > 0,05; Zusatzmaterial online: Tab. 2).

### Ausmaß des Belastungserlebens

Über die verschiedenen Beobachtungszeitpunkte (t0–t4) ergab sich kein signifikanter Haupteffekt in Bezug auf das Belastungserleben der TN mittels BS oder Mini-SCL sowie auf die Frequenz der Telefonkontakte (alle *p* > 0,05; Zusatzmaterial online: Tab. 3). Ein signifikanter Haupteffekt zeigte sich für die Person sowohl für die BS als auch für die Mini-SCL (BS: *p* < 0,01; Mini-SCL: *p* < 0,05); Angehörige waren im Durchschnitt über alle Zeitpunkte hinweg stärker belastet als Patient:innen. Das durchschnittliche Beschwerdeerleben lag bei allen TN im unauffälligen Bereich, Angehörige erreichten jedoch insgesamt höhere Werte (Mini-SCL-Rohwerte: Pat.: 1,75–4,89; Ang.: 5,75–8,5). Das subjektive Belastungserleben bewegte sich bei Patient:innen im Bereich zwischen keiner und leichter (1,22–2,0) Belastung, bei Angehörigen zwischen leichter und mäßiger (2,73–3,73) Belastung (Zusatzmaterial online: Tab. 4). Signifikante Interaktionseffekte ließen sich weder zwischen Person • Beobachtungszeitpunkten noch zwischen Gruppe • Beobachtungszeitpunkten noch hinsichtlich Person • Gruppe • Beobachtungszeitpunkte nachweisen (alle *p* > 0,05, Zusatzmaterial online: Tab. 4).

Für die differenzierte Angehörigenbelastung (BIZA-D-PV) ließ sich kein signifikanter Haupteffekt über den Beobachtungszeitraum oder den Faktor Gruppe ermitteln (alle *p* > 0,05). Ein signifikanter Interaktionseffekt Gruppe • Zeit lässt sich nur für die Subskala D Veränderungen Kognition (*p* < 0,05) ermitteln (Zusatzmaterial online: Tab. 3). Im Mittel waren die Angehörigen in der Erfassung auf allen Skalen (A–E) mäßig belastet (Zusatzmaterial online: Tab. 5).

### Akzeptanz und Bewertung der Überbrückungsmaßnahmen

Der Abschlussfragebogen wurde von 42 TN zurückgesendet und zeigt, dass die Überbrückungsmaßnahmen von den meisten TN positiv angenommen wurden. Fünf (12,2 %) TN bewerteten die Maßnahmen insgesamt als wenig bis nicht hilfreich; zwei TN beendeten die telefonischen Kontakte vorzeitig (Drop-out: 4,7 %). Im Vergleich der Gruppen ließen sich bis auf einen signifikanten Effekt bei der Frage, ob die regelmäßigen Telefongespräche als hilfreich empfunden wurden (*p* < 0,05; Cramers V = 0,36 = mittelgradiger Effekt; wöchentlich: 91,7 %; 14-tägig: 88,3 %), keine weiteren signifikanten Unterschiede ermitteln. Somit werden im Folgenden ausschließlich Häufigkeiten berichtet. Als Zustimmung werden die Ausprägungen „trifft eher zu“ und „trifft voll zu“, als Ablehnung „trifft eher nicht zu“ und „trifft gar nicht zu“ interpretiert (Zusatzmaterial online: Tab. 6).

Als belastend wurde die COVID-19-Pandemie von insgesamt 33 (78,6 %) TN empfunden, wobei Angehörige eher zustimmten (90 % vs. 68,2 %). Über zwei Drittel der TN (71,4 %) bestätigten, dass ihnen während der COVID-19-Pandemie soziale Kontakte gefehlt hätten, Angehörige waren etwas stärker betroffen (Pat.: 63,7 %; Ang.: 80 %). Auch vermissten mehr Angehörige den COVID-19-bedingten Wegfall von Hobbys (Ang.: 65,0 %; Pat.: 45,5 %). Explizit nach dem Ausfall von Aktiv+++ befragt, gaben 80 % der Befragten an, dass ihnen der regelmäßige Besuch gefehlt hätte (Pat.: 86,4 %; Ang.: 72,2 %).

Die telefonische Begleitung wurde von 90,3 % der TN als hilfreich empfunden (Ang.: 100 %; Pat: 81 %). Zudem gaben 83,4 % der Befragten an, dass die Telefongespräche ihrem/ihrer (Ehe‑)Partner:in gutgetan hätten, was auch den Großteil der Telefonate (78 %) mit stärker demenziell beeinträchtigen Patient:innen betrifft. In einer vergleichbaren Situation würden 85,7 % der TN eine telefonische Begleitung erneut in Anspruch nehmen wollen.

Bei 92,1 % der befragten TN wurde die Zusendung von Aufgaben positiv bewertet (Ang.: 100 %, Pat.: 86,4 %), zudem würden 86,9 % der TN (Ang.: 93,8 %; Pat.: 81,8 %) die Zusendung von Aufgaben in einer vergleichbaren Situation wieder in Anspruch nehmen wollen. Abschließend gaben 87,8 % der TN an, dass ihnen die Maßnahmen während der COVID-19-bedingten Einschränkungen geholfen hätten (Ang.: 94,8 %, Pat.: 81,9 %).

## Diskussion

Zur Unterstützung bei COVID-19-bedingter Aussetzung des ambulanten psychosozialen Behandlungsprogramms Aktiv+++ im Frühjahr 2020 erhielten alle TN eine telefonische Begleitung, Patient:innen wurden zusätzlich kognitive und körperliche Aktivierungsaufgaben zugesendet. Gegenstände der vorliegenden Behandlungsbeobachtung waren die Überprüfung der Akzeptanz der Überbrückungsmaßnahmen sowie die Erfassung des Belastungserlebens der TN. Überdies war von Interesse, ob die Distanzmaßnahmen eine Behandlungsoption therapeutischer Angebote für nichtpandemieassoziierte Umstände zur Verbesserung der Versorgung demenziell Erkrankter und deren Angehörigen sein könnten.

Grundsätzlich stellte es eine große Herausforderung dar, innerhalb von 14 Tagen ein Überbrückungsprogramm zu konzipieren, welches die stadienspezifischen Ressourcen bzw. Defizite der Patient:innen berücksichtigt und dieses über mehrere Wochen durchzuführen. Zudem ist hervorzuheben, dass – entgegen vieler Untersuchungsdesigns mit demenziell Erkrankten – auch von Patient:innen mit beginnender Demenz selbstbeurteilende Angaben erfasst wurden.

Die regelmäßigen Telefonkontakte sowie die Auswahl und Zusendung von Aufgaben per Post über den Beobachtungszeitraum waren gut durchführbar. Insgesamt wurden die Überbrückungsmaßnahmen sowohl von Patient:innen als auch von deren Angehörigen positiv angenommen, was sich durch die geringe Drop-out-Rate zeigte. Hinsichtlich der Akzeptanz können die Überbrückungsangebote somit positiv bewertet werden.

Im Hinblick auf das Belastungserleben waren Angehörige signifikant stärker belastet als Patient:innen, was möglicherweise auf den Wegfall von Dienstleistungen und Hilfestrukturen während der COVID-19-bedingten Einschränkungen zurückzuführen ist [8]. Gleichzeitig kann das bei den Patient:innen geringer ausgeprägte Belastungserleben auf ein reduziertes Urteilsvermögen in Bezug auf Belastungsfaktoren oder eine verringerte Krankheitswahrnehmung als Erklärung diskutiert werden. Die Frequenz der Telefonkontakte hatte auf das allgemeine Belastungserleben keinen Einfluss, allerdings bewerteten die TN – hier explizit die Angehörigen – die wöchentlichen Telefonkontakte als hilfreicher im Vergleich zu 14-tägigen Kontakten. In Anbetracht des relativ hohen Gesamtaufwands mit wöchentlichen Kontakten und der geringfügig günstigeren Bewertung gegenüber den 14-tägigen Kontakten ergibt sich aus pragmatischer Sicht eine Präferenz für 14-tägige Kontakte bei zukünftigen Unterstützungsangeboten.

Das leicht ausgeprägte Belastungserleben der TN veränderte sich über dem Beobachtungszeitraum nicht wesentlich. Dies ist möglicherweise dadurch zu erklären, dass ältere Menschen ihre Resilienz, basierend auf krisenbezogenen Erfahrungskontexten, im Vergleich zu jüngeren als höher einschätzen und damit funktionaler mit der Pandemie umgehen konnten [10]. Hinweise auf eine ausgeprägte psychische Widerstandkraft ergeben sich aus einer telefonischen Befragung, die zeigt, dass die mentale und soziale Gesundheit bei über 65-Jährigen während eines 4‑wöchigen „Lockdowns“ im April 2020 nahezu unverändert blieb [14]. Da der Großteil der Befragten angab, die Angebote als hilfreich empfunden zu haben, könnten aber auch die Überbrückungsmaßnahmen dazu beigetragen haben. Hierfür lassen sich auch in anderen Untersuchungsergebnissen Hinweise finden [10]. Dennoch kann die Frage, welche Faktoren oder Maßnahmen ausschlaggebend und wie nachhaltig diese für eine stabile psychische Gesundheit unter Pandemiebedingungen sind, nur mit systematischen Untersuchungen unter Berücksichtigung spezifischer demografischer und klinischer Subgruppen mit ausgedehnten Beurteilungszeiträumen beantwortet werden.

Hinsichtlich des Belastungserlebens der Angehörigen in Bezug auf die kognitiven Veränderungen des zu Betreuenden und der Frequenz der telefonischen Begleitung zeigte sich über den Untersuchungszeitraum bei den Angehörigen mit wöchentlichen Kontakten eine Belastungsreduktion im Gegensatz zu denjenigen mit 14-tägigen Kontakten. Ein möglicher Erklärungsansatz ist, dass die hochfrequent kontaktierten Angehörigen durch die engmaschigere Unterstützung und der im Rahmen der Telefonate vermittelten psychoedukativen und metakognitiven Inhalte in Bezug auf demenzielle Erkrankungen mit den kognitiven Veränderungen der Patient:innen besser umgehen konnten. Allerdings stellt sich die Frage, warum sich positive Effekte auf das Belastungserleben der wöchentlich kontaktierten Angehörigen nicht auch in anderen Subskalen niedergeschlagen haben. Ein diesbezüglicher Trend zeigt sich auch unter Einbeziehung deskriptiver Daten (Zusatzmaterial online: Tab. 5) nicht.

Zusammenfassend ist zu sagen, dass die Überbrückungsmaßnahmen für einen COVID-19-bedingten Ausfall eines ambulanten Behandlungsprogramms für demenziell Erkrankte und deren Angehörige von den TN gut angenommen und positiv bewertet wurden. Das subjektive Belastungserleben der TN veränderte sich im Untersuchungszeitraum nicht, was möglicherweise auf intrinsische Faktoren, wie z. B. eine hohe Resilienz, zurückzuführen ist. Ob und inwieweit Interventionsansätze, die frühestmöglich zum Einsatz kommen, einen Beitrag leisten, um negative Auswirkungen für die psychosoziale Gesundheit verringern zu können, kann mit der vorliegenden Behandlungsbeobachtung nicht abschließend beantwortet werden. Hierzu wären randomisierte kontrollierte Untersuchungen mit größeren Stichproben und längeren Nachuntersuchungszeiträumen erforderlich.

Die positiven Erfahrungen mit den Überbrückungsmaßnahmen könnten auch im Hinblick auf die Forderung der „Nationalen Demenzstrategie“ (NDS) einen Beitrag leisten, neue Versorgungsformen in der Routineversorgung für demenziell Erkrankte und deren Angehörige zu entwickeln und zu überprüfen. „Distanz-Angebote“ könnten Betroffene, deren Teilhabe z. B. aufgrund von Mobilitätseinschränkungen oder Leben im ländlichen Raum ohne Zentrumsanbindung erschwert bzw. nicht möglich ist, Zugang zu und Inanspruchnahme von ambulanten psychosozialen Behandlungsangeboten erleichtern und damit die Versorgungssituation verbessern [3].

## Limitationen

Mit der vorliegenden explorativen Behandlungsbeobachtung können nur unmittelbare Einschätzungen und Bewertungen nach Teilnahme an den Überbrückungsmaßnahmen erfasst werden. Eine Aussage über die Dauerhaftigkeit von Beobachtungen wie z. B. im Hinblick auf das subjektive Belastungserleben kann somit nicht getroffen werden. Zudem ist eine Generalisierung der Beobachtungen vor dem Hintergrund der stark selektierten Stichprobe kaum möglich. Aufgrund der teilweise kleinen Subgruppen und der damit begrenzten statistischen Aussagekraft ist eine differenzierte Betrachtung von möglichen Unterschieden in Bezug auf den Schweregrad der demenziellen Erkrankung wenig möglich gewesen. Aussagen zur Wirksamkeit der gewählten Überbrückungsmaßnahmen sind bei fehlender randomisierter Kontrollgruppe überdies nicht möglich.

## Fazit für die Praxis

- Pandemiebedingte Schutzmaßnahmen mit weitreichenden Ausgangs- und Kontaktbeschränkungen können bei demenziell erkrankten Menschen und deren Angehörigen soziale Teilhabechancen mindern, was negative psychosoziale Folgen haben kann.
- Überbrückungsmaßnahmen für demenziell erkrankte Menschen und deren Angehörige in Form regelmäßiger telefonischer Kontakte mit zusätzlichen per Post zugeschickten Aufgaben zu geistiger und körperlicher Aktivierung sind mit entsprechender personeller Unterstützung gut durchführbar und werden von den Betroffenen gerne in Anspruch genommen.
- Erfahrungen mit COVID-19-bedingten Überbrückungsmaßnahmen befürworten einen erneuten Einsatz im Falle einer zukünftigen pandemiebedingten Behandlungsaussetzung und können darüber hinaus im Sinne der „Nationalen Demenzstrategie“ zur Entwicklung neuer Versorgungsformen für demenziell Erkrankte und deren Angehörige genutzt werden, um die Inanspruchnahme vormals ambulanter Behandlungsangebote zu ermöglichen.

## Supplementary Information




