# Supplementary material for: Acceptance of psychosocial bridging measures in dementia: Treatment observation during the COVID-19 pandemic
Source: Z Gerontol Geriatr. 2022 Oct 7;56(7):573–9. [Article in German] doi: 10.1007/s00391-022-02115-6 (PMC9540087; doi:10.1007/s00391-022-02115-6)
Supplement: Supplementary file 1 [file 391_2022_2115_MOESM1_ESM.docx]

**Tabelle  1** Demografische und klinische Daten der Teilnehmer:innen (TN) zu Beginn der Behandlungsbeobachtung (n=45)

| **Variable** | **Gruppe A (wöchentliche Telefonkontakte)** | | **Gruppe A gesamt**  **(n=24)** | **Gruppe B (14-tägige Telefonkontakte)** | | **Gruppe B**  **gesamt (n=21)** | **Gesamtstichprobe**  **(n=45)** | |
| --- | --- | --- | --- | --- | --- | --- | --- | --- |
|  | Pat.  (n=12) | Ang.  (n=12) | gesamt | Pat.  (n=12) | Ang.  (n=9) | gesamt |  | |
|  | **MW (+/-SD)** | | | | | | | |
| **Alter** | 80,3 (6,9) | 76,3 (7,4) | 78,3 (7,3) | 79,2 (7,3) | 73,0 (12,0) | 76,6 (9,8) | 77,5 (8,5) | |
| **Belastungserleben** | 1,8 (0,9) | 3,7 (1,2) | 2,8 (1,4) | 1,6 (0,8) | 2,8 (1,1) | 2,2 (1,1) | 2,5 (1,3) | |
|  | Pat.  (n=12) | Ang.  (n=12) | gesamt | Pat.  (n=12) | Ang.  (n=9) | gesamt |  | |
| **Geschlecht** | **abs. (in %)** | | | | | | | |
| weiblich | 4 (16,7) | 9 (37,5) | 13 (54,2) | 5 (23,8) | 7 (33,3) | 12 (57,1) | 25 (55,6) | |
| männlich | 8 (33,3) | 3 (12,5) | 11 (45,8) | 7 (33,3) | 2 (9,5) | 9 (42,9) | 20 (44,4) | |
| **Familienstand** |  | | | | | | | |
| verheiratet | 8 (33,3) | 12 (50,0) | 20 (83,3) | 9 (42,9) | 9 (42,9) | 18 (85,7) | 38 (84,4) | |
| geschieden | 1 (4,2) | 0 (0,0) | 1 (4,2) | 0 (0,0) | 0 (0,0) | 0 (0,0) | 1 (2,2) | |
| verwitwet | 3 (12,5) | 0 (0,0) | 3 (12,5) | 3 (14,3) | 0 (0,0) | 3 (14,3) | 6 (13,3) | |
| **Bildungsstand** | **abs. (in %)** | | | | | | | |
| ohne Abschluss | 1 (4,2) | 0 (0) | 1 (4,2) | 0 (0,0) | 0 (0,0) | 0 (0,0) | 1 (2,2) | |
| Hauptschulabschluss | 1 (4,2) | 2 (8,3) | 3 (12,5) | 1 (4,8) | 2 (9,5) | 3 (14,3) | 6 (13,3) | |
| Realschulabschluss | 2 (8,3) | 4 (16,7) | 6 (25,0) | 4 (19,0) | 5 (23,8) | 9 (42,9) | 15 (33,3) | |
| Fachhochschulreife | 1 (4,2) | 0 (0,0) | 1 (4,2) | 0 (0,0) | 0 (0,0) | 0 (0,0) | 1 (2,2) | |
| Allgemeine Hochschulreife | 7 (29,2) | 6 (25,0) | 13 (54,2) | 7 (33,3) | 2 (9,5) | 9 (42,9) | 22 (48,9) | |
| **Wohnsituation** | **abs. (in %)** | | | | | | | |
| Haus/Wohnung mit Garten | 9 (37,5) | 9 (37,5) | 18 (75,0) | 11 (52,4) | 8 (38,1) | 19 (90,5) | 37 (82,2) | |
| Wohnung mit Terrasse/Balkon | 3 (12,5) | 3 (12,5) | 6 (25,0) | 0 (0,0) | 1 (4,8) | 1 (4,8) | 7 (15,6) | |
| Wohnung | 0 (0,0) | 0 (0,0) | 0 (0,0) | 1 (4,8) | 0 (0,0) | 1 (4,8) | 1 (2,2) | |
| **Schweregrad*** | **abs. (in %)** | | | | | | **Gesamt Pat. (n=24)** | **Gesamt Ang.**  **(n=21)** |
| Leichte kognitive Störung (MCI) | 4 (16,7) | 2 (9,5) |  | 6 (25,0) | 2 (9,5) |  | 10 (41,7) | 4 (19,0) |
| leichtgradige Demenz | 8 (33,3) | 5 (23,8) |  | 6 (25,0) | 3 (14,3) |  | 14 (58,3) | 8 (38,1) |
| mittelgradige Demenz |  | 3 (14,3) |  |  | 2 (9,5) |  |  | 5 (23,8) |
| mittel- bis schwergradige Demenz |  | 2 (9,5) |  |  | 2 (9,5) |  |  | 4 (19,0) |

MW= Mittelwert; SD= Standardabweichungen; * bezieht sich bei den Angehörigen auf den Schweregrad des/der zu betreuenden/pflegenden Patient:in

Pat.= Patient:innen; Ang.= Angehörige

**Tabelle 2** Unterschiede zwischen Gruppen (Frequenz telefonischer Kontakte) zu Beginn der Behandlungsbeobachtung

|  | **Gesamtstichprobe**  **Gruppe A vs Gruppe B** |  |  |
| --- | --- | --- | --- |
| **Variable** |  | **Patient:innen**  **Gruppe A vs Gruppe B** | **Angehörige**  **Gruppe A vs Gruppe B** |
| Alter | *t*=0,67; *p*=0,51 | *t*=0,35; *p*=0,73 | *t*=0,78; *p*=0,44 |
| Belastungserleben (BS) | *t*=10,51; *p*=0,14 | *t*=0,61; *p*=0,55 | *t*=10,79; *p*=0,09 |
| Geschlecht | χ^2^=0,04; *p*=0,84 | χ^2^=0,18; *p*=0,67* | χ^2^=0,02; *p*=0,88* |
| Familienstand | χ^2^ =10,29; *p*=0,52* | χ^2^=10,45; *p*=0,49* | alle n verheiratet |
| Bildungsstand | χ^2^ =30,91; *p*=0,42* | χ^2^ =30,45; *p*=0,49* | χ^2^=10,77; *p*=0,41* |
| Wohnsituation | χ^2^ =50,18; *p*=0,08* | χ^2^=50,75; *p*=0,06* | χ^2^=0,68; *p*=0,41* |
| Schweregrad** |  | χ^2^=0,69; *p*=0,41* | χ^2^=0,28; *p*=0,96* |

* Likelihood-ratio-Statistik; ** bezieht sich bei den Angehörigen auf den Schweregrad des/der zu betreuenden/pflegenden Patient:in

**Tabelle 3** Übersicht der Varianzanalyse mit abhängigen (BS und Mini-SCL) und unabhängigen (Gruppenzugehörigkeit und Person) Zielvariablen

| **Zeit** |  |  |
| --- | --- | --- |
|  | BS** | *F*(3.40,105.24)=1.58, *p*=.19, *η^2^=.05* |
|  | Mini-SCL*** | *F*(2.98,92.32)=0.71, *p*=.55, *η^2^=.02* |
| **Gruppe** |  |  |
|  | BS** | F(1,31)=1.62, p=.21, *η^2^=.05* |
|  | Mini-SCL*** | F(1,31)=2.56, p=.12, *η^2^=.08* |
| **Person****** |  |  |
|  | BS** | F(1,31)=32.22, p<.01**, *η^2^=.51* |
|  | Mini-SCL*** | F(1,31)=7.21, *p*<.05**, η^2^=.19* |
| **Gruppe x Zeit** |  |  |
|  | BS** | *F(3.4,105.24)=0.26, p=.88* |
|  | Mini-SCL*** | F(2.98,92.32)=0.34, p=.80 |
| **Person x Zeit** |  |  |
|  | BS** | *F*(3.4,105.24)=0.14, *p*=.95 |
|  | Mini-SCL*** | F(2.98,92.32)=1.74, p=.17 |
| **Gruppe x Person** |  |  |
|  | BS** | F(1,31)=0,41, p=.53, *η^2^=.01* |
|  | Mini-SCL*** | F(1,31)=.05, p=.83, *η^2^=.01* |
| **Person x Gruppe x Zeit** |  |  |
|  | BS** | *F*(3.4,105.24)=1.89, *p*=.13, *η^2^=.06* |
|  | Mini-SCL*** | *F*(2.98,92.32) F=.13, *p*=.94, *η^2^=.00* |

* zweifaktorielle Varianzanalyse; **Belastungserleben (BS); ***Mini-Symptom-Checklist (Mini-SCL)

**** Patient:in versus Angehörige/r

**Tabelle 4** Übersicht Belastungserleben (BS) und Beschwerdeerleben (Mini-SCL) nach Person (Patient:innen/Angehörige) und Gruppenzugehörigkeit (Frequenz telefonischer Kontakte; n=35)

| **Variable**  **Zeitpunkt** | **Belastungserleben (BS)** | | **Beschwerdeerleben (Mini-SCL)** | |
| --- | --- | --- | --- | --- |
|  | **Pat.**  **Gruppe A vs Gruppe B** | **Ang.**  **Gruppe A vs Gruppe B** | **Pat.**  **Gruppe A vs Gruppe B** | **Ang.**  **Gruppe A vs Gruppe B** |
| t0 | 1,80 (.92) vs. 1,56 (.88) | 3,73 (1.19) vs. 2,75 (1.17) | 4,89 (3.76) vs. 2,0 (1.85) | 8,5 (6,3) vs. 5,87 (4.67) |
| t1 | 2,0 (1.05) vs. 1,33 (.50) | 3,09 (1.04) vs. 2,75 (1.17) | 5,22 (4.35) vs. 2,63 (2.0) | 6,1 (3,07) vs. 5,37 (3.62) |
| t2 | 2,30 (1,52) vs. 1,33 (.71) | 3,13 (1.27) vs. 3,13 (.99) | 5,78 (5.74) vs. 3,38 (4.6) | 7,4 (7,78) vs. 5,75 (3.45) |
| t3 | 2,20 (1,14) vs. 1,33 (.71) | 3,27 (1.04) vs. 3,13 (.99) | 3,78 (4.21) vs. 1,13 (2.1) | 9,2 (9.33) vs. 6,63 (4.10) |
| t4 | 2,0 (1.25) vs. 1,22 (.67) | 2,73 (.65) vs. 2,88 (.99) | 2,78 (3.15) vs. 1,75 (2.49) | 7,0 (5.89) vs. 5,75 (3.37) |

*es werden Mittelwerte **+/-** Standardabweichungen ( ) berichtet; Pat.= Patient:innen; Ang.= Angehörige

**Tabelle 5** Übersicht Angehörigenbelastung (BIZA-D-PV) nach Gruppenzugehörigkeit (Frequenz telefonischer Kontakte: n=12)

|  | **Berliner Inventar zur Angehörigenbelastung-Demenz-Manual zur Praxisversion (BIZA-D-PV)** | | | | |
| --- | --- | --- | --- | --- | --- |
| **t** | **A: Persönliche Einschränkungen**  **Gruppe A vs Gruppe B** | **B: Mangelnde soziale Unterstützung**  **Gruppe A vs Gruppe B** | **C: Akzeptieren der Situation**  **Gruppe A vs Gruppe B** | **D: kognitive Veränderungen**  **Gruppe A vs Gruppe B** | **E: Verhaltensveränderungen**  **Gruppe A vs Gruppe B** |
| t0 | 9,92 (±4.48) vs. 8,29 (±2.56) | 3,67 (±3.0) vs. 4,0 (±2.71) | 12,75 (±3.17) vs. 12,29 (±2.81) | 9,17 (±4,45) vs. 8,43 (±2.3) | 5,25 (±4,6) vs. 2,14 (±2.48) |
| t4 | 7,58 (±6.55) vs. 8,29 (±3.2) | 3,83 (±4.65) vs. 3,14 (±3.13) | 13,08 (±2.88) vs. 13,0 (±1.29) | 7,50 (±4.8) vs. 9,57 (±3,95) | 4,75 (±4.69) vs. 2,86 (±2.85) |

* es werden Mittelwerte ± Standardabweichungen ( ) berichtet

**Tabelle 6** Abschließende Bewertung von Corona-bedingten Veränderungen und Bewertung der Überbrückungsmaßnahmen

| **Themen und Fragen aus dem Abschlussfragebogen** | **Gruppe** | **trifft gar nicht zu**  abs./in% | **trifft eher nicht zu**  abs./in% | **trifft eher zu**  abs./in% | **trifft voll zu**  abs./in% |
| --- | --- | --- | --- | --- | --- |
| **Beurteilung Corona-bedingter Veränderungen** |  |  |  |  |  |
| Die Corona-Pandemie habe ich bisher als belastend empfunden. | A (n=24) | 1 (4,2) | 4 (16,7) | 13 (54,2) | 6 (25,0) |
|  | B (n=18) | 0 (0,0) | 4 (22,2) | 7 (38,9) | 7 (38,9) |
|  | ges. n=42 | 1 (2,4) | 8 (19,0) | 20 (47,6) | 13 (31,0) |
| Soziale Kontakte haben mir während der Corona-bedingten Einschränkungen in den vergangenen Wochen gefehlt. | A (n=24) | 3 (12,5) | 6 (25,0) | 10 (41,7) | 5 (20,8) |
|  | B (n=18) | 1 (5,6) | 2 (11,1) | 6 (33,3) | 9 (50,0) |
|  | ges. 42 | 4 (9,5) | 8 (19,0) | 16 (38,1) | 14 (33,3) |
| Hobbies ausüben zu können, wie z.B. Sport oder kulturelle Unternehmungen, habe ich während der Corona-bedingten Einschränkungen in den vergangenen Wochen vermisst. | A (n=24) | 6 (25,0) | 6 (25,0) | 7 (29,2) | 5 (20,8) |
|  | B (n=18) | 2 (11,1) | 5 (27,8) | 4 (22,2) | 7 (38,9) |
|  | ges. 42 | 8 (19,0) | 11 (26,2) | 11 (26,2) | 12 (28,6) |
| Mir ist bei meiner (Ehe-)Partnerin/meinem (Ehe-)Partner in den vergangenen Wochen eine negative Veränderung aufgefallen. | A (n=20) | 4 (20,0) | 1 (5,0) | 12 (60,0 | 3 (15,0) |
|  | B (n=14) | 4 (28,6) | 3 (21,4) | 4 (28,6) | 3 (21,4) |
|  | ges. 34 | 8 (23,5) | 4 (11,8) | 16 (47,1) | 6 (17,6) |
| Die schon vor der Corona-Pandemie bestehenden Verände-rungen meiner (Ehe-)Partnerin/meines (Ehe-)Partners haben mich während der vergangenen Wochen maßgeblich belastet. | A (n=12) | 1 (8,3) | 2 (16,7) | 7 (58,3) | 2 (16,7) |
|  | B (n=7) | 2 (28,6) | 1 (14,3) | 3 (42,9) | 1 (14,3) |
|  | ges. 19 | 3 (15,8) | 3 (15,8) | 10 (52,6) | 3 (15,8) |
| **Bewertung der Überbrückungsmaßnahmen** |  |  |  |  |  |
| Die regelmäßigen Telefongespräche mit den Mitarbeiterinnen des Memory-Zentrums habe ich als hilfreich empfunden. | A (n=24) | 0 (0,0) | 2 (8,3) | 9 (37,5) | 13 (54,2) |
|  | B (n=17) | 0 (0,0) | 2 (11,8) | 1 (5,9) | 14 (82,4) |
|  | ges. 41 | 0 (0,0) | 4 (9,8) | 10 (24,4) | 27 (65,9) |
| Die telefonische Begleitung würde ich in einer vergleichbaren Situation wieder in Anspruch nehmen. | A (n=24) | 2 (8,3) | 2 (8,3) | 5 (20,8) | 15 (62,5) |
|  | B (n=18) | 1 (5,6) | 1 (5,6) | 3 (16,7) | 13 (72,2) |
|  | ges. 42 | 3 (7,1) | 3 (7,1) | 8 (19,0) | 28 (66,7) |
| Dass mir regelmäßig Aufgaben zur geistigen und körperlichen Aktivierung per Post zugeschickt wurden, fand ich gut. | A (n=19) | 1 (5,3) | 1 (5,3) | 4 (21,1) | 13 (68,4) |
|  | B (n=14) | 0 (0,0) | 1 (5,9) | 1 (5,9) | 12 (70,6) |
|  | ges. 33 | 1 (2,8) | 2 (5,6) | 5 (13,9) | 25 (69,4) |
| Die Aufgaben per Post würde ich in einer vergleichbaren Situation wieder in Anspruch nehmen wollen. | A (n=19) | 1 (5,3) | 3 (15,8) | 3 (15,8) | 12 (63,2) |
|  | B (n=14) | 0 (0,0) | 1 (5,9) | 1 (5,9) | 12 (70,6) |
|  | ges. 33 | 1 (2,8) | 4 (11,1) | 4 (11,1) | 24 (66,7) |
| Der regelmäßige Besuch der Aktiv+++-Gruppen hat mir während der Corona-bedingten Einschränkungen der vergangenen Wochen gefehlt. | A (n=23) | 2 (8,7) | 4 (17,4) | 5 (21,7) | 12 (52,2) |
|  | B (n=17) | 0 (0,0) | 2 (11,8) | 3 (17,6) | 12 (70,6) |
|  | ges. 40 | 2 (5,0) | 6 (15,0) | 8 (20,0) | 24 (60,0) |
| **Fremdbeurteilung** |  |  |  |  |  |
| Die regelmäßigen Telefongespräche mit meiner (Ehe-) Partnerin/meinem (Ehe-) Partner mit den Mitarbeiterinnen des Memory-Zentrums haben ihr/ihm gutgetan. | A (n=19) | 0 (0,0) | 4 (21,1) | 11 (57,9) | 4 (21,1) |
|  | B (n=11) | 0 (0,0) | 1 (9,1) | 3 (27,3) | 7 (63,6) |
|  | ges. 30 | 0 (0,0) | 5 (16,7) | 14 (46,7) | 11 (36,7) |
| Dass meiner (Ehe-)Partnerin/meinem (Ehe-)Partner regelmäßig Aufgaben zur geistigen und körperlichen Aktivierung per Post zugeschickt wurden, fand ich gut** | Ang. (n=16) | 0 (0,0) | 0 (0,0) | 3 (18,8) | 13 (81,3) |
| Aufgaben per Post würde ich für meine (Ehe-)Partnerin/meinen (Ehe-) Partner in einer vergleichbaren Situation wieder in Anspruch nehmen wollen. | Ang. (n=16) | 0 (0,0) | 1 (6,3) | 2 (12,5) | 13 (81,3) |
| **Abschließende Bewertung** |  |  |  |  |  |
| Die Maßnahmen des Memory-Zentrums (telefonische Begleitung, Aufgaben per Post) haben mir bei der Bewältigung während der Corona-bedingten Einschränkungen geholfen. | A (n=24) | 1 (4,2) | 2 (8,3) | 10 (41,7) | 11 (45,8) |
|  | B (n=17) | 1 (5,9) | 1 (5,9) | 4 (23,5) | 11 (64,7) |
|  | ges. 41 | 2 (4,9) | 3 (7,3) | 14 (34,1) | 22 (53,7) |

* Pat.= Patient:innen; Ang.=Angehörige **Im Hinblick auf die Bewertung der regelmäßig per Post zugesandten (Trainings-)Unterlagen wurde der Eigenbewertung der Patient:innen auch die der Angehörigen zur Seite gestellt, um die Akzeptanz aller Beteiligten zu erfassen.
